# On the application, reporting, and sharing of in silico simulations for genetic studies

**DOI:** 10.1002/gepi.22362

**Published:** 2020-10-16

**Authors:** Kaleigh Riggs, Huann‐Sheng Chen, Melissa Rotunno, Bing Li, Naoko I. Simonds, Leah E. Mechanic, Bo Peng

**Affiliations:** ^1^ Department of Statistics Rice University Houston Texas USA; ^2^ Division of Cancer Control and Population Sciences, Statistical Research and Applications Branch, Surveillance Research Program, National Cancer Institute (NCI) National Institutes of Health (NIH) Bethesda Maryland USA; ^3^ Division of Cancer Control and Population Sciences, Genomic Epidemiology Branch Epidemiology and Genomics Research Program, NCI, NIH Bethesda Maryland USA; ^4^ Department of Biostatistics Brown University Providence Rhode Island USA; ^5^ Naoko Ishibe Simonds Consulting Englewood New Jersey USA; ^6^ Department of Medicine Baylor College of Medicine Houston Texas USA

**Keywords:** data sets, Genetic simulations, reproducibility

## Abstract

In silico simulations play an indispensable role in the development and application of statistical models and methods for genetic studies. Simulation tools allow for the evaluation of methods and investigation of models in a controlled manner. With the growing popularity of evolutionary models and simulation‐based statistical methods, genetic simulations have been applied to a wide variety of research disciplines such as population genetics, evolutionary genetics, genetic epidemiology, ecology, and conservation biology. In this review, we surveyed 1409 articles from five journals that publish on major application areas of genetic simulations. We identified 432 papers in which genetic simulations were used and examined the targets and applications of simulation studies and how these simulation methods and simulated data sets are reported and shared. Whereas a large proportion (30%) of the surveyed articles reported the use of genetic simulations, only 28% of these genetic simulation studies used existing simulation software, 2% used existing simulated data sets, and 19% and 12% made source code and simulated data sets publicly available, respectively. Moreover, 15% of articles provided no information on how simulation studies were performed. These findings suggest a need to encourage sharing and reuse of existing simulation software and data sets, as well as providing more information regarding the performance of simulations.

## 1 INTRODUCTION

In silico genetic simulations—or computer modeling of genetic data under specified assumptions—and the software tools used to generate these simulations (which we define as genetic simulators) have played an important role in the development and applications of statistical methods for genetic studies in multiple disciplines (Daetwyler et al., 2013; Epperson et al., 2010; Hoban et al., 2012; Peng et al., 2015). These simulations have been used for different applications, such as to validate statistical methods with model‐specific assumptions (Zheng et al., 2019); to test the robustness of methods against deviations from model assumptions (Skotte et al., 2019); to estimate the Type‐I error, sensitivity (Chen et al., 2018), specificity, and power (Zhao et al., 2018) of statistical methods using a large number of replicated simulations; to compare the performance between multiple methods (Ramstetter et al., 2018; Zhou et al., 2017); to infer parameters of statistical models from simulations that best match empirical data (Oaks et al., 2019); and to explore outcomes of evolutionary processes under varying assumptions (Mooney et al., 2018).

Genetic simulations vary greatly in the types of data being simulated (which we will refer to as targets of simulations), methods and tools used to simulate data or certain processes, and in the roles that they play in genetic studies. Samples simulated under the null hypothesis (e.g., genotypes that are unrelated to a disease) can be used to evaluate how likely a method would identify a wrong signal (Type‐1 error), and samples simulated under the alternative hypothesis (e.g., genotypes that are related to a disease) can be used to evaluate how likely a method can successfully detect a true signal (empirical power). Simulations can also be performed to explore parameter space with no assumptions of an underlying hypothesis (exploratory simulations), or to infer parameters of their hypothesis‐based models by comparing simulated data sets with observed ones (statistical inference). In silico simulations can be used to validate different analytical methods. For example, samples that are simulated according to assumptions of a statistical model can be used to validate if the model works under its assumed conditions (validation of statistical models), or samples that are simulated under nonconforming assumptions (e.g., different statistical distributions of input data and the presence of missing data or outliers) can be used to test the robustness of a method, or samples that mimic properties of real‐world data sets (therefore independent of model assumptions) can be used to test validity and performance of statistical models in real‐world applications. Finally, applications of simulations have been important in the development of statistical methods, where they have been used to test the validity of the method, evaluate Type‐1 error, power, robustness, sensitivity and specificity, and perhaps most importantly, compare the performance of a novel method with existing ones.

Different applications of genetic simulations demand different designs and implementations. For example, simulations designed to validate a statistical method need to follow the assumptions of the method closely and often employ model‐based simulations, whereas simulations designed to test real‐world performance need to follow the properties observed in real data. The latter typically use sideway methods (i.e., a method that generates simulated data sets by using existing data and resampling it) and is also preferred for comparing performance across multiple statistical methods as it is not as likely to bias toward any particular method being compared. Due to these different requirements, the level of sophistication of genetic simulations inevitably varies (Dasgupta et al., 2011; Engelman et al., 2016; Fragoulakis et al., 2019), and dedicated computer programs have been developed to perform genetic simulations and generate simulated data sets designed with different modeling emphases (Peng et al., 2013).

To obtain a better understanding of how genetic simulations are being generated, used, reported, and shared, we conducted a review of the literature. In this review, we provide details from 423 articles from five journals that publish on major application areas of genetic simulations, such as, epidemiology, bioinformatics, biomedical discoveries, and evolution theory. In addition, we examined whether simulations were performed using pre‐existing software and whether the data and/or code were publicly available.

## 2 METHODS

We performed a review of the literature to examine the approaches used for genetic simulations. We focused on in silico simulations that used computers to generate “hypothetical” data, manipulated empirically collected data sets, such as those from genetic epidemiological studies, or used genetic computer simulations in other genetics studies, spanning a variety of disciplines related to genetics.

### 2.1 Selection of journals

To select articles for this review, we identified all the journals that were included as either “citations” or “applications” in the Genetic Simulation Resource (GSR) catalog (Peng et al., 2013). These journals included *American Journal of Human Genetics (AJHG), Bioinformatics, BMC Bioinformatics, BMC Genomics, Evolution, Frontiers in Genetics, Genetic Epidemiology, Genetics, Hereditary, Molecular Biology and Evolution, Molecular Ecology Resources, Nature Communication, Nature Genetics, PLoS Genetics, PLoS One, Proceedings of Biological Sciences, Proceedings of the National Academy Sciences of the United States of America*, and *Scientific Reports*. First, we selected one recent issue from each of the 19 journals and recorded the number of articles that reported using simulations. Next, we selected the five journals with the highest proportion of articles that included simulated data within distinct scientific topic areas (Table S1). For instance, *BMC Bioinformatics* and *Bioinformatics* both reported a relatively high fraction of articles using genetic simulations, but we randomly selected only *Bioinformatics* because of overlap in the content area with *BMC Bioinformatics*.

The five journals that we ultimately selected for detailed analysis were *AJHG, Bioinformatics, Evolution, Genetic Epidemiology*, and *Nature Genetics*. These journals represent major scientific areas of genetic simulations such as epidemiology, bioinformatics, biomedical research, and evolution theory. We searched these journals starting from the end date of July 2019 and reviewed all previous issues until we identified either 100 articles using simulated data (for journals with a large number of simulation papers) or up to 2 years of articles for journals that had published fewer than 100 articles using genetic simulations. This strategy was used to ensure a review of a sufficient, roughly equal number of articles to make meaningful conclusions.

### 2.2 Selection of articles

We defined an article to contain simulated data if the data were generated completely using in silico models or simulated from real data or parameters. We excluded studies that used empirical data sets (e.g., 1000 genomes; Auton et al., 2015) to validate or compare statistical methods although the difference between simulated and empirical data sets can be subtle. For example, a simulation study could ascertain random samples from empirical data sets, and some empirical data sets could be computationally enhanced (e.g., imputed against reference genome). We define simulation studies as the use of computers to manipulate data by the authors and consider random ascertainment of empirical data as simulations. Targets of simulations included genetic data (e.g., genetic markers and DNA sequences, RNA sequences and gene expressions, protein sequences, and protein abundances), observed characteristics associated with genetic data (e.g., qualitative and quantitative traits), the relationship between genetic data (e.g., pathways, networks, and phylogenetic trees), and mathematical models that describe genetic data. Other targets of simulations included were cells (type, shape, growth, etc.), drugs, and treatment effects, among others.

### 2.3 Data collected

We abstracted information from the articles using simulated data, summarized in Table S2. The information collected included: date of publication, target of simulation, application of simulation, and how simulations were conducted and reported. Any individual article may contain multiple simulations; therefore, several characteristics were not mutually exclusive. We consider a simulation to be developed using in‐house methods if the script or software was developed by the article authors without using existing simulation software or simulated data sets, independent of whether such a method was later made publicly available. Other information abstracted included how the software was used, whether the simulations were conducted using particular software, and whether and where the data and the code for the simulation are available. Over 50% of the articles were quality controlled by a second reviewer.

We classify applications of simulations into five categories (Summary Box 1). We report multiple applications for articles that use more than one simulation studies for multiple purposes (e.g. for both validation and performance comparison).

SUMMARY BOX 1 **Categories of applications of simulations**

**Table:** 

Application	Characteristics
Estimation of Type 1 error, sensitivity, specificity, or statistical power	Application of statistical methods on multiple replicates of simulated data sets to quantify success rate of detecting signals (or no signal) in the data sets
Validation of correctness or robustness of models	Application of statistical methods on data sets simulated according to or deviate from specific model assumptions
Statistical inference	Simulations of data with varying parameters and the identification of simulations (and therefore parameters) that best match empirical data
Comparison of performance of statistical methods	Application of multiple statistical methods on the same set of simulated data sets for the purposes of comparing results
Exploratory simulations	Exploration of results from simulations with varying parameters to observe possible outcomes associated with a parameter space

## 3 RESULTS

We included for review 423 articles with simulations from 1409 total articles surveyed from the five selected journals. The complete abstracted data are provided as supplementary material 2B. *Genetic Epidemiology* published the highest proportion of articles that utilize genetic simulations (72.4%), followed by *Evolution* (35.7%), *Bioinformatics* (31.6%), *Nature Genetics* (22.5%), and *AJHG* (12.4%; Table 1). Some characteristics were similar across all five journals. For example, the frequency of articles that reported using pre‐existing methods ranged 25.6%–31.0%. However, other characteristics, such as the proportion of articles that provided details on the simulated data sets or provided source codes, varied by a journal. Specifically, out of the 423 articles, 49 (11.6%) provided publicly available simulated data sets and 82 (19.4%) provided source codes. When this data was examined by journal, most of these articles were from the journal *Evolution*; almost half of the articles reported details of simulations in the format of source code (*N* = 47) and almost a third of the articles provided simulated data sets (*N* = 28), in contrast to the other four journals.

**Table 1:** Summary of articles using genetic simulations surveyed from five selected journals

	Genetic Epidemiology	Bioinformatics	American Journal of Human Genetics	Nature Genetics	Evolution	Total
Total articles surveyed	134	277	354	364	280	1409
Articles with genetic simulations^a^	97 (72.4%)	100 (31.6%)	44 (12.4%)	82 (22.5%)	100 (35.7%)	423 (30.0%)
Among the articles with genetic simulations and abstracted						
Articles that perform simulations with pre‐existing software	27 (27.8%)	26 (26.0%)	12 (27.2%)	21 (25.6%)	31 (31.0%)	117 (27.7%)
Articles that make simulated data set publicly available^b^	2 (2.1%)	13 (13.0%)	2 (4.5%)	4 (4.9%)	28 (28.5%)	49 (11.6%)
Articles that make source code for simulations available^b^	2 (2.1%)	18 (18.0%)	6 (13.6%)	9 (11.0%)	47 (47.0%)	82 (19.4%)
Articles that make use of existing simulated data sets	0 (0%)	7 (7%)	0 (0%)	0 (0%)	1 (1%)	8 (1.9%)

a For each of the five journals, we surveyed either 100 articles with genetic simulations or all articles with genetic simulations in the past 2 years. We were able to survey 100 articles in the journals of Bioinformatics and Evolution, but fewer than 100 articles from the other three journals.

b The numbers include data sets and source code that were not immediately available (embargoed) but were available upon request. We did not contact the authors to confirm the availability of such data or source code.

### 3.1 Methods used to conduct simulations

Even though 67 (15%) articles provided little to no information on how the simulation studies were performed, most articles described the methods used. The majority of the articles reported using in‐house simulation methods (72% of 423), and the proportions were similar across the five journals (from 69% in *Evolution* to 74% in *Nature Genetics*). Many of the in‐house methods were implemented using general‐purpose scripting languages such as R, Python, and MATLAB and only onefifth were made publicly available.

The remaining 117 (27%) articles (ranging from 12 in *AHSG* to 31 in *Evolution*, Table 1) reported using an existing simulator, which are listed in Table S2. Overall, COSI (*N* = 13, of which 11 in *Genetic Epidemiology*;Schaffner et al., 2005 *)*, HapGen2 (*N* = 8, all in *Genetic Epidemiology*; Su et al., 2011), and SLiM (*N* = 6, of which 4 in *Nature Genetics*; Haller & Messer, 2017, 2019) were the most commonly used simulators for the simulation of genotype data in this sample of articles, whereas PhyTools (Revell, 2012; *N* = 11, all in *Evolution*) was the most popular for the simulation of phylogenetic trees. Other tools were used sparsely for other specific applications, including GeneNetWeaver (Schaffter et al., 2011; *N* = 3 in *Bioinformatics*), DWGSIM, and ART (Huang et al., 2012; *N* = 2 in *Bioinformatics*). Other tools were only used once each (Huang et al., 2012; Schaffter et al., 2011).

Eight of the 423 articles (1.8% or 8 of 423, of which 7 in *Bioinformatics*, Table 1) used pre‐existing simulated data sets. Out of the eight, three used DREAM Challenges data (Cao et al., 2019; Ghanbari et al., 2019; Zheng et al., 2019), one used the CAMI2 simulated human microbiome data sets (Shi et al., 2019), and the remaining four used simulated data sets from previously published papers (Caetano et al., 2018; Han et al., 2019; Kim et al., 2019; Yu et al., 2018).

### 3.2 Sharing of simulation programs and data sets

Among 49 articles with publicly available simulated data (11.3%), most simulated data sets were saved to repositories such as DRYAD (*N* = 22), GitHub (*N* = 6), GitBuckets (*N* = 1), FigShare (*N* = 1), and UniShare.nl (*N* = 1). Others were provided as supplementary material (*N* = 3), posted on authors’ personal websites (*N* = 2), and one was available upon request. The size of shared simulated data sets varied greatly, with the largest data sets saved to UniShare.nl (53 GB; Xu & Etienne, 2018) and DRYAD (23 GB; Culshaw et al., 2019 and 18 GB; Hvala et al., 2018). Similarly, among 82 articles with publicly available source codes for simulation studies, most were saved to code sharing platforms: 29 to DRYAD, 29 to GitHub, 2 to FigShare, 1 to GitBucket, and 1 to Zenodo. The source code was available as supplementary material in five articles and available upon request in two articles.

### 3.3 Targets and applications of simulations

Overall, genetic simulations have simulated a wide array of data and processes (targets), played many roles (applications) in genetic studies, and the distributions of targets and applications varied from journal to journal (Tables 2 and 3). Many studies simulated multiple targets for multiple applications. For example, many genetic epidemiological studies simulated both genotype and phenotype data estimated the power (and Type‐I error, etc.) of a new genetic association test, and compared its performance with existing methods.

**Table 2:** Number^a^ and proportion^b^ of articles by targets of genetic simulations by journal

Target of genetic simulation^c^	Genetic Epidemiology	Bioinformatics	American Journal of Human Genetics	Nature Genetics	Evolution
Genotype	88 (90.7%)	71 (71.0%)	32 (72.7%)	64 (78.0%)	20 (20.0%)
Phenotype	87 (89.7%)	30 (30.0%)	17 (38.6%)	36 (42.7%)	24 (24.0%)
Relationship	35 (36.1%)	21 (21.0%)	13 (29.5%)	16 (19.5%)	32 (32.0%)
Mathematical models	5 (5.2%)	11 (11.0%)	8 (20.5%)	10 (12.2%)	11 (11.0%)
Others	1 (1.0%)	8 (8.0%)	2 (4.5%)	5 (6.1%)	39 (39.0%)
Total number of articles reviewed	**97**	**100**	**44**	**82**	**100**

a Number of articles does not add up to the total due to multiple types of data simulated in each article.

b Proportion of articles do not add to 100%.

c Target of genetic simulation are defined in Section 2.

**Table 3:** Number^a^ and proportion^b^ of articles by applications of genetic simulations by journal

Applications of genetic simulation^c^	Genetic Epidemiology	Bioinformatics	American Journal of Human Genetics	Nature Genetics	Evolution
Type‐1 error, power, and sensitivity	68 (70.1%)	23 (23.0%)	19 (43.2%)	30 (36.6%)	9 (9.0%)
Comparison of statistical methods performance	84 (86.6%)	69 (69.0%)	20 (45.5%)	21 (25.6%)	4 (4.0%)
Validation/robustness	31 (32.0%)	50 (50.0%)	13 (29.5%)	33 (40.2%)	19 (19.0%)
Exploratory analysis	3 (3.1%)	0 (0%)	2 (4.5%)	5 (6.1%)	32 (32.0%)
Statistical inference	0 (0%)	2 (2.0%)	10 (22.7%)	24 (29.3%)	57 (57.0%)
Total number of articles reviewed	**97**	**100**	**44**	**82**	**100**

a Number of articles does not add up to the total due to genetic simulations being used for multiple applications.

b Proportion of articles do not add to 100%.

c Definitions for applications of genetic simulation are provided in Section 2.

The targets of simulation studies reflected the scope of the articles that the journals publish. As the field of genetic epidemiology focuses largely on deciphering genetic causes of human diseases and traits, it is not surprising that 90% of simulation studies in this journal simulated genotypes and phenotypes. Upon closer examination, 86.4% of these articles simulated DNA (markers, sequences, and sequencing reads) while the remainder simulated RNA and other types of genetic data such as gene expression (see Table S2 for details). Compared with *Genetic Epidemiology*, the journals *Bioinformatics, AJHG*, and *Nature Genetics* place less emphasis on phenotypes and included a broader range of genetic studies. Although genotypes remained the most common targets for simulations performed in these journals, around half of the studies published simulated RNA and corresponding expressions, microbiome, summary statistics, and annotations. A large portion of articles in *Evolution* simulated phylogenetic trees to study the relationship among species and genes. Articles in this journal also simulated targets that are less commonly studied in the other four journals, such as mating patterns and seed banking strategies (McCullough et al., 2018).

Likewise, the types of applications varied by journal, where 70% of articles in *Genetic Epidemiology* used genetic simulations to evaluate the performance of statistical methods. The proportion of published articles that evaluated the performance of statistical methods were substantially lower in the other journals, especially *Evolution*. Similarly, we observed differences in the percentages of papers published by journal that compared the performance of existing statistical methods. For this application type, *Bioinformatics* published the largest proportion of such articles. This finding is consistent with its aims where studies published in *Bioinformatics* often focus on identifying the best methods to address specific bioinformatic challenges, rather than on developing new methods. Finally, not surprisingly, simulations used for the purposes of statistical inference were most frequently observed in *Evolution* followed by *Nature Genetics*, as the aim of these journals is to draw conclusions from genetic data.

## 4 DISCUSSION

We surveyed 1409 articles from journals and identified 423 (30%) articles that utilized genetic simulations. Based on our abstraction of these 423 articles, we observed less than onethird of these publications reported using pre‐existing simulation methods and only eight used pre‐existing data sets. About 15% of articles provided no information about how simulations were performed. In addition, the proportion of articles that shared data (11.6%) or source code (19.4%) was limited. We also observed variability across journals in the proportion of articles that utilized genetic simulations, where 72% of articles in the journal of *Genetic Epidemiology* used genetic simulations compared with 12.4% of articles in *AJHG*. Furthermore, we observed differences in patterns of targets and applications that were simulated across the journals, likely reflecting differences in the aim and scope of each journal. For example, 70% of the articles in *Genetic Epidemiology* that reported using genetic simulations were for the validation and comparison of performance of statistical methods compared with journals that focus more on applications of genetic studies, such as *AJHG* and *Nature Genetics*.

Approximately 70% of the publications surveyed used in‐house genetic simulations methods, of which only around 20% were made publicly available. Although it is too early to predict how many of these in‐house methods will be reused in the future, we observed that only three articles from our review used in‐house methods previously published (Mak et al., 2017; Wainberg et al., 2019; Wang et al., 2017), possibly indicating that most in‐house methods are rarely reused. A large number of in‐house simulation methods and their limited sharing and reuse are likely due to multiple reasons. Some of the genetic studies used simple simulations for one‐time use only; these were based on general‐purpose scripting languages, such as the use of random number generators to generate phenotypes with certain distributions or random sampling process to ascertain samples from a pool of data sets. Researchers may prefer to develop their own in‐house methods to more readily control all aspects relevant to the specific research requirements (Chen et al., 2015). Unlike data sets generated carefully by organizations such as the Genetic Analysis Workshop (Blangero et al., 2016) and DREAM (Lee et al., 2018; Marbach et al., 2012), simulations designed for a specific research paper often have nonstandard formats, less documentation, and are difficult to reuse. Researchers may also choose to create their own simulation code rather than search for available software especially when the simulations are not very complicated. In addition, simulations created for other statistical methods are likely based on specific assumptions that provide important performance information for those scenarios, and they may not be suitable for evaluating or comparing the power of methods based on different assumptions.

Additional reasons that may have contributed to the underutilization of existing tools include a lack of detailed description of simulations (e.g., source code and parameters used) and the unavailability of simulated data sets leading to the high proportion of in‐house simulations observed. The National Cancer Institute created the GSR website with the intent of increasing awareness of existing tools; however, more needs to be done to address the availability of data and source code (Chen et al., 2015; Peng et al., 2013). Reusing existing data and software would maximize the utility of existing resources and should be encouraged to limit potential replication of effort and redundancy of tools. Comparison and evaluation of different simulators are also needed to identify the strengths and weaknesses of available methods (Chen et al., 2015). Furthermore, creating in‐house methods that are tailored to answer a particular research question may lead to an optimistic interpretation of results (Chen et al., 2015; Mechanic et al., 2012). For example, in genetic simulation of rare genetic variants, many models assume a large percentage of highly penetrant mutations, resulting in optimistic power assumptions. Therefore, the use of common simulation programs or data sets has been encouraged. Due to the wide range of applications for which genetic simulations can be used, it is not feasible to have a single dedicated simulation program for all possible applications and the development of new simulation methods may be necessary.

Among the five journals surveyed, articles published in *Evolution* most consistently deposited simulated data sets and related source code. Although Figshare and some other (e.g., university) file‐sharing mechanisms were occasionally used, almost all articles published in *Evolution* deposited data in DRYAD (Khan & Weeks, 2015), which is a data repository funded by multiple publishers and supported by multiple institutions. We attribute this observation to the *Evolution* journal's policies, which states “Authors must make their empirical raw data and analytic methods available to other researchers and must specify where that material is available.” Moreover, its guidelines to authors explicitly require, “as a condition for publication, that data supporting the results in the paper should be archived in an appropriate public archive, such as DRYAD, Figshare, GenBank, TreeBASE, the Knowledge Network for Biocomplexity, or other suitable long‐term and stable public repositories.” The other four journals surveyed did not explicitly require authors to share data or source codes, which may account for part of the differences observed. The policy adopted by *Evolution* originated from a Transparency and Openness Promotion framework that contains editorial guidelines for journals. The purpose of this policy is to help achieve data and material transparency such that other researchers can replicate the procedure and reproduce results (Nosek et al., 2015). It is highly recommended that disciplines such as genetics and bioinformatics follow these guidelines.

There are several public repositories available for the deposit of research data sets and source code. Although some, like DRYAD, collect a service fee or is subscription based (which is often paid by institutions), services such as Figshare and Zenodo are available free of charge (with some restrictions). Due to the popularity of Git repository hosting services, websites such as GitHub and BitBucket are frequently used to store source code, and, in some cases, simulated data sets (da Fonseca et al., 2019). However, source code repositories are not designed for the storage of data sets and have restrictions on file size (e.g., GitHub limits files to those less than 100 MB). In addition, these general‐purpose data repositories may exacerbate the reusability problem by accepting a wide range of data types in a wide variety of formats (Wilkinson et al., 2016). Previously, in some instances, simulated data sets have been deposited to the National Institutes of Health (NIH)‐funded repositories such as dbGaP (e.g., simulated data sets for the Framingham Heart Study; Cupples et al., 2009). For simulated data sets not generated from identifiable human subjects, other alternative NIH repositories without requirements for controlled access could be explored to promote the sharing of these data sets.

In addition to public repositories, other common ways for storing data and source code related to research articles include personal, institutional, and journal websites. Compared with public repositories that are designed for the storage, display, and retrieval of research data for a prolonged period of time, data stored on personal, institutional, and journal websites are less organized and sometimes inaccessible. For example, the website that hosts data and code for one of the articles was temporarily unavailable during our review. For these reasons, journals often recommend using public repositories for the deposit of research code and data.

This survey has some limitations. One limitation is that the survey was restricted to five journals that were selected for inclusion. Narrowly defining the inclusion criteria provides only a snapshot of the field. However, the included journals represent major application areas of genetic simulations and our observations are consistent with what has been reported previously, suggesting that expanding the scope of journals would not substantially change our results (Peng et al., 2015). Another limitation is that we did not evaluate the in‐house methods to see whether the simulations could be performed, and hence, could not determine the quality of the description of the in‐house methods. This is an important open question that should be addressed in future studies.

A large number of papers using genetic simulations for a variety of purposes and the limited observed use of existing simulation tools/data sets as well as sharing of these, suggest the need for best practices for reporting and sharing of these data sets, as was discussed previously (Chen et al., 2015). Although efforts have been made to facilitate the sharing of data sets and software, additional efforts are needed by journals and funding agencies to encourage the reuse of these resources to extract maximum benefit from research investments. We have summarized the key findings and suggest recommendations based on these findings to facilitate research in this area (Summary Box 2). Because genetic simulations are essential for the study of genetics of complex diseases, greater sharing of simulated data sets and related source code could help accelerate the development and application of statistical methods for genetic studies in multiple disciplines benefiting the scientific community.

SUMMARY BOX 2 **Key messages and recommendations**

**Key messages**

- 30% of articles in surveyed journals utilized genetic simulations, depending on discipline and journal (range, 12.4%–72.0%).
- 15% of articles provided no information on how the simulation studies were performed.
- 70% of articles surveyed used in‐house simulation methods. About 28% of articles used existing simulation software and 2% used existing simulated data sets.
- 12% of articles made simulated data sets and 19% of articles made source code for simulation studies publicly available.

**Recommendations based on these findings**

- The scientific community should encourage reuse of existing simulation software and/or data sets to facilitate fair comparison between statistical methods and to maximize benefit from research investment.
- Research articles should report complete information on how simulations were performed.
- Source code and simulated data sets should be deposited and shared in public repositories to facilitate reuse and reproducibility.
- Journal offices and funding agencies could encourage proper reporting and sharing of simulation studies by establishing guidelines and policies.

## CONFLICT OF INTERESTS

The authors declare that there are no conflict of interests.

## Supporting information

Supporting information.

## Data Availability

The data that supports the findings of this study are available in the supplementary material of this article.

## References

[gepi22362-bib-0001] Auton, A. , Abecasis, G. R. , Altshuler, D. M. , Durbin, R. M. , Abecasis, G. R. , Bentley, D. R. , Chakravarti, A. , Clark, A. G. , Donnelly, P. , Eichler, E. E. , Flicek, P. , Gabriel, S. B. , Gibbs, R. A. , Green, E. D. , Hurles, M. E. , Knoppers, B. M. , Korbel, J. O. , Lander, E. S. , Lee, C. , … Abecasis, G. R. , The 1000 Genomes Project Consortium . (2015). A global reference for human genetic variation. Nature, 526(7571), 68–74. 26432245 10.1038/nature15393 PMC4750478

[gepi22362-bib-0002] Blangero, J. , Teslovich, T. M. , Sim, X. , Almeida, M. A. , Jun, G. , Dyer, T. D. , Johnson, M. , Peralta, J. M. , Manning, A. , Wood, A. R. , Fuchsberger, C. , Kent, J. W. , Aguilar, D. A. , Below, J. E. , Farook, V. S. , Arya, R. , Fowler, S. , Blackwell, T. W. , Puppala, S. , … Almasy, L. (2016). Omics‐squared: Human genomic, transcriptomic and phenotypic data for genetic analysis workshop 19. BMC Proceedings, 10(Suppl 7), 71–77. 27980614 10.1186/s12919-016-0008-y PMC5133484

[gepi22362-bib-0003] Caetano, D. S. , O'Meara, B. C. , & Beaulieu, J. M. (2018). Hidden state models improve state‐dependent diversification approaches, including biogeographical models. Evolution, 72(11), 2308–2324. 30226270 10.1111/evo.13602

[gepi22362-bib-0004] Cao, C. , Mak, L. , Jin, G. , Gordon, P. , Ye, K. , & Long, Q. (2019). PRESM: Personalized reference editor for somatic mutation discovery in cancer genomics. Bioinformatics, 35(9), 1445–1452. 30247633 10.1093/bioinformatics/bty812

[gepi22362-bib-0005] Chen, H. S. , Hutter, C. M. , Mechanic, L. E. , Amos, C. I. , Bafna, V. , Hauser, E. R. , Hernandez, R. D. , Li, C. , Liberles, D. A. , McAllister, K. , Moore, J. H. , Paltoo, D. N. , Papanicolaou, G. J. , Peng, B. , Ritchie, M. D. , Rosenfeld, G. , Witte, J. S. , Gillanders, E. M. , & Feuer, E. J. (2015). Genetic simulation tools for post‐genome wide association studies of complex diseases. Genetic Epidemiology, 39(1), 11–19. 25371374 10.1002/gepi.21870 PMC4270837

[gepi22362-bib-0006] Chen, P. , Jing, X. , Ren, J. , Cao, H. , Hao, P. , & Li, X. (2018). Modelling BioNano optical data and simulation study of genome map assembly. Bioinformatics, 34(23), 3966–3974. 29893801 10.1093/bioinformatics/bty456 PMC6247929

[gepi22362-bib-0007] Culshaw, V. , Stadler, T. , & Sanmartin, I. (2019). Exploring the power of Bayesian birth‐death skyline models to detect mass extinction events from phylogenies with only extant taxa. Evolution, 73(6), 1133–1150. 31017656 10.1111/evo.13753 PMC6767073

[gepi22362-bib-0008] Cupples, L. A. , Heard‐Costa, N. , Lee, M. , & Atwood, L. D. (2009). Genetics analysis workshop 16 problem 2: The Framingham heart study data. BMC Proceedings, 3(Suppl 7), S3. 10.1186/1753-6561-3-s7-s3 PMC2795927 20018020

[gepi22362-bib-0009] Daetwyler, H. D. , Calus, M. P. L. , Pong‐Wong, R. , de los Campos, G. , & Hickey, J. M. (2013). Genomic prediction in animals and plants: Simulation of data, validation, reporting, and benchmarking. Genetics, 193(2), 347–65. 23222650 10.1534/genetics.112.147983 PMC3567728

[gepi22362-bib-0010] Dasgupta, A. , Sun, Y. V. , König, I. R. , Bailey‐Wilson, J. E. , & Malley, J. D. (2011). Brief review of regression‐based and machine learning methods in genetic epidemiology: The Genetic Analysis Workshop 17 experience. Genetic Epidemiology, 35(Suppl 1), S5–S11. 22128059 10.1002/gepi.20642 PMC3345521

[gepi22362-bib-0011] Engelman, C. D. , Greenwood, C. M. T. , Bailey, J. N. , Cantor, R. M. , Kent, J. W. , König, I. R. , Bermejo, J. L. , Melton, P. E. , Santorico, S. A. , Schillert, A. , Wijsman, E. M. , MacCluer, J. W. , & Almasy, L. (2016). Genetic Analysis Workshop 19: Methods and strategies for analyzing human sequence and gene expression data in extended families and unrelated individuals. BMC Proceedings, 10(Suppl 7), 67–70. 27980613 10.1186/s12919-016-0007-z PMC5133501

[gepi22362-bib-0012] Epperson, B. K. , Mcrae, B. H. , Scribner, K. , Cushman, S. A. , Rosenberg, M. S. , Fortin, M. J. , James, P. M. A. , Murphy, M. , Manel, S. , Legendre, P. , & Dale, M. R. T. (2010). Utility of computer simulations in landscape genetics. Molecular Ecology, 19(17), 3549–64. 20618894 10.1111/j.1365-294X.2010.04678.x

[gepi22362-bib-0013] da Fonseca, N. J. , Afonso, M. Q. L. , de Oliveira, L. C. , & Bleicher, L. (2019). A new method bridging graph theory and residue co‐evolutionary networks for specificity determinant positions detection. Bioinformatics, 35(9), 1478–1485. 30295749 10.1093/bioinformatics/bty846

[gepi22362-bib-0014] Fragoulakis, V. , Roncato, R. , Fratte, C. D. , Ecca, F. , Bartsakoulia, M. , Innocenti, F. , Toffoli, G. , Cecchin, E. , Patrinos, G. P. , & Mitropoulou, C. (2019). Estimating the Effectiveness of DPYD genotyping in Italian individuals suffering from cancer based on the cost of chemotherapy‐induced toxicity. American Journal of Human Genetics, 104(6), 1158–1168. 31155283 10.1016/j.ajhg.2019.04.017 PMC6557730

[gepi22362-bib-0015] Ghanbari, M. , Lasserre, J. , & Vingron, M. (2019). The distance precision matrix: Computing networks from non‐linear relationships. Bioinformatics, 35(6), 1009–1017. 30165509 10.1093/bioinformatics/bty724 PMC6420154

[gepi22362-bib-0016] Haller, B. C. , & Messer, P. W. (2017). SLiM 2: Flexible, interactive forward genetic simulations. Molecular Biology and Evolution, 34(1), 230–240. 27702775 10.1093/molbev/msw211

[gepi22362-bib-0017] Haller, B. C. , & Messer, P. W. (2019). SLiM 3: Forward genetic simulations beyond the Wright‐Fisher model. Molecular Biology and Evolution, 36(3), 632–637. 30517680 10.1093/molbev/msy228 PMC6389312

[gepi22362-bib-0018] Han, R. , Wan, X. , Li, L. , Lawrence, A. , Yang, P. , Li, Y. , Wang, S. , Sun, F. , Liu, Z. , Gao, X. , & Zhang, F. (2019). AuTom‐dualx: A toolkit for fully automatic fiducial marker‐based alignment of dual‐axis tilt series with simultaneous reconstruction. Bioinformatics, 35(2), 319–328. 30010792 10.1093/bioinformatics/bty620 PMC6330008

[gepi22362-bib-0019] Hoban, S. , Bertorelle, G. , & Gaggiotti, O. E. (2012). Computer simulations: Tools for population and evolutionary genetics. Nature Reviews Genetics, 13(2), 110–22. 10.1038/nrg3130 22230817

[gepi22362-bib-0020] Huang, W. , Li, L. , Myers, J. R. , & Marth, G. T. (2012). ART: A next‐generation sequencing read simulator. Bioinformatics, 28(4), 593–594. 22199392 10.1093/bioinformatics/btr708 PMC3278762

[gepi22362-bib-0021] Hvala, J. A. , Frayer, M. E. , & Payseur, B. A. (2018). Signatures of hybridization and speciation in genomic patterns of ancestry. Evolution, 72, 1540–1552. 10.1111/evo.13509 PMC6261709 29806154

[gepi22362-bib-0022] Khan, K. , & Weeks, A. D. (2015). Example of retrospective dataset publication through Dryad. BMJ, 350, h1788. 25873188 10.1136/bmj.h1788

[gepi22362-bib-0023] Kim, C. S. , Mohan, S. , Ayub, M. , Rothwell, D. G. , Dive, C. , Brady, G. , & Miller, C. (2019). In silico error correction improves cfDNA mutation calling. Bioinformatics, 35(14), 2380–2385. 30520956 10.1093/bioinformatics/bty1004 PMC6612818

[gepi22362-bib-0024] Lee, A. Y. , Ewing, A. D. , Ellrott, K. , Hu, Y. , Houlahan, K. E. , Bare, J. C. , Espiritu, S. M. G. , Huang, V. , Dang, K. , Chong, Z. , Caloian, C. , Yamaguchi, T. N. , Kellen, M. R. , Chen, K. , Norman, T. C. , Friend, S. H. , Guinney, J. , Stolovitzky, G. , Haussler, D. , … Boutros, P. C. (2018). Combining accurate tumor genome simulation with crowdsourcing to benchmark somatic structural variant detection. Genome Biology, 19(1), 188. 10.1186/s13059-018-1539-5 30400818 PMC6219177

[gepi22362-bib-0025] Mak, T. S. H. , Porsch, R. M. , Choi, S. W. , Zhou, X. , & Sham, P. C. (2017). Polygenic scores via penalized regression on summary statistics. Genetic Epidemiology, 41(6), 469–480. 28480976 10.1002/gepi.22050

[gepi22362-bib-0026] Marbach, D. , Costello, J. C. , Küffner, R. , Vega, N. M. , Prill, R. J. , Camacho, D. M. , Allison, K. R. , Kellis, M. , Collins, J. J. , & Stolovitzky, G. (2012). Wisdom of crowds for robust gene network inference. Nature Methods, 9(8), 796–804. 22796662 10.1038/nmeth.2016 PMC3512113

[gepi22362-bib-0027] McCullough, E. L. , Buzatto, B. A. , & Simmons, L. W. (2018). Population density mediates the interaction between pre‐ and postmating sexual selection. Evolution, 72(4), 893–905. 29455461 10.1111/evo.13455

[gepi22362-bib-0028] Mechanic, L. E. , Chen, H. S. , Amos, C. I. , Chatterjee, N. , Cox, N. J. , Divi, R. L. , Fan, R. , Harris, E. L. , Jacobs, K. , Kraft, P. , Leal, S. M. , McAllister, K. , Moore, J. H. , Paltoo, D. N. , Province, M. A. , Ramos, E. M. , Ritchie, M. D. , Roeder, K. , Schaid, D. J. , … Gillanders, E. M. (2012). Next generation analytic tools for large scale genetic epidemiology studies of complex diseases. Genetic Epidemiology, 36(1), 22–35. 22147673 10.1002/gepi.20652 PMC3368075

[gepi22362-bib-0029] Mooney, J. A. , Huber, C. D. , Service, S. , Sul, J. H. , Marsden, C. D. , Zhang, Z. , Sabatti, C. , Ruiz‐Linares, A. , Bedoya, G. , Freimer, N. , Lohmueller, K. E. , Fears, S. C. , Service, S. K. , Kremeyer, B. , Lic, C. A. , Lic, X. A. , Bejarano, J. , Lic, M. R. , Castrillón, G. , … Coppola, G. (2018). Understanding the hidden complexity of Latin American population isolates. American Journal of Human Genetics, 103(5), 707–726. 30401458 10.1016/j.ajhg.2018.09.013 PMC6218714

[gepi22362-bib-0030] Nosek, B. A. , Alter, G. , Banks, G. C. , Borsboom, D. , Bowman, S. D. , Breckler, S. J. , Buck, S. , Chambers, C. D. , Chin, G. , Christensen, G. , Contestabile, M. , Dafoe, A. , Eich, E. , Freese, J. , Glennerster, R. , Goroff, D. , Green, D. P. , Hesse, B. , Humphreys, M. , … Yarkoni, T. (2015). Promoting an open research culture. Science, 348(6242), 1422–1425. 26113702 10.1126/science.aab2374 PMC4550299

[gepi22362-bib-0031] Oaks, J. R. , Siler, C. D. , & Brown, R. M. (2019). The comparative biogeography of Philippine geckos challenges predictions from a paradigm of climate‐driven vicariant diversification across an island archipelago. Evolution, 73(6), 1151–1167. 31017301 10.1111/evo.13754 PMC6767427

[gepi22362-bib-0032] Peng, B. , Chen, H. S. , Mechanic, L. E. , Racine, B. , Clarke, J. , Clarke, L. , Gillanders, E. , & Feuer, E. J. (2013). Genetic simulation resources: A website for the registration and discovery of genetic data simulators. Bioinformatics, 29(8), 1101–1102. 23435068 10.1093/bioinformatics/btt094 PMC3624809

[gepi22362-bib-0033] Peng, B. , Chen, H. S. , Mechanic, L. E. , Racine, B. , Clarke, J. , Gillanders, E. , & Feuer, E. J. (2015). Genetic data simulators and their applications: An overview. Genetic Epidemiology, 39(1), 2–10. 25504286 10.1002/gepi.21876 PMC4804465

[gepi22362-bib-0034] Ramstetter, M. D. , Shenoy, S. A. , Dyer, T. D. , Lehman, D. M. , Curran, J. E. , Duggirala, R. , Blangero, J. , Mezey, J. G. , & Williams, A. L. (2018). Inferring identical‐by‐descent sharing of sample ancestors promotes high‐resolution relative detection. American Journal of Human Genetics, 103(1), 30–44. 29937093 10.1016/j.ajhg.2018.05.008 PMC6035284

[gepi22362-bib-0035] Revell, L. J. (2012). phytools: An R package for phylogenetic comparative biology (and other things). Methods in Ecology and Evolution, 3(2), 217–223.

[gepi22362-bib-0036] Schaffner, S. F. (2005). Calibrating a coalescent simulation of human genome sequence variation. Genome Research, 15(11), 1576–83. 16251467 10.1101/gr.3709305 PMC1310645

[gepi22362-bib-0037] Schaffter, T. , Marbach, D. , & Floreano, D. (2011). GeneNetWeaver: In silico benchmark generation and performance profiling of network inference methods. Bioinformatics, 27(16), 2263–70. 21697125 10.1093/bioinformatics/btr373

[gepi22362-bib-0038] Shi, L. , Meng, X. , Tseng, E. , Mascagni, M. , & Wang, Z. (2019). SpaRC: Scalable sequence clustering using Apache Spark. Bioinformatics, 35(5), 760–768. 30816928 10.1093/bioinformatics/bty733

[gepi22362-bib-0039] Skotte, L. , Jørsboe, E. , Korneliussen, T. S. , Moltke, I. , & Albrechtsen, A. (2019). Ancestry‐specific association mapping in admixed populations. Genetic Epidemiology, 43(5), 506–521. 30883944 10.1002/gepi.22200

[gepi22362-bib-0040] Su, Z. , Marchini, J. , & Donnelly, P. (2011). HAPGEN2: Simulation of multiple disease SNPs. Bioinformatics, 27(16), 2304–2305. 21653516 10.1093/bioinformatics/btr341 PMC3150040

[gepi22362-bib-0041] Wainberg, M. , Sinnott‐Armstrong, N. , Mancuso, N. , Barbeira, A. N. , Knowles, D. A. , Golan, D. , Ermel, R. , Ruusalepp, A. , Quertermous, T. , Hao, K. , Björkegren, J. L. M. , Im, H. K. , Pasaniuc, B. , Rivas, M. A. , & Kundaje, A. (2019). Opportunities and challenges for transcriptome‐wide association studies. Nature Genetics, 51(4), 592–599. 30926968 10.1038/s41588-019-0385-z PMC6777347

[gepi22362-bib-0042] Wang, K. , Gaitsch, H. , Poon, H. , Cox, N. J. , & Rzhetsky, A. (2017). Classification of common human diseases derived from shared genetic and environmental determinants. Nature Genetics, 49(9), 1319–1325. 28783162 10.1038/ng.3931 PMC5577363

[gepi22362-bib-0043] Wilkinson, M. D. , Dumontier, M. , Aalbersberg, I. J. , Appleton, G. , Axton, M. , Baak, A. , Blomberg, N. , Boiten, J. W. , da Silva Santos, L. B. , Bourne, P. E. , Bouwman, J. , Brookes, A. J. , Clark, T. , Crosas, M. , Dillo, I. , Dumon, O. , Edmunds, S. , Evelo, C. T. , Finkers, R. , … Mons, B. (2016). The FAIR guiding principles for scientific data management and stewardship. Scientific Data, 3, 160018. 26978244 10.1038/sdata.2016.18 PMC4792175

[gepi22362-bib-0044] Xu, L. , & Etienne, R. S. (2018). Detecting local diversity‐dependence in diversification. Evolution, 72(6), 1294–1305. 29626347 10.1111/evo.13482 PMC6055638

[gepi22362-bib-0045] Yu, G. , Jiang, Y. , Wang, J. , Zhang, H. , & Luo, H. (2018). BMC3C: Binning metagenomic contigs using codon usage, sequence composition and read coverage. Bioinformatics, 34(24), 4172–4179. 29947757 10.1093/bioinformatics/bty519

[gepi22362-bib-0046] Zhao, N. , Zhan, X. , Huang, Y. T. , Almli, L. M. , Smith, A. , Epstein, M. P. , Conneely, K. , & Wu, M. C. (2018). Kernel machine methods for integrative analysis of genome‐wide methylation and genotyping studies. Genetic Epidemiology, 42(2), 156–167. 29285792 10.1002/gepi.22100

[gepi22362-bib-0047] Zheng, R. , Li, M. , Chen, X. , Wu, F. X. , Pan, Y. , & Wang, J. (2019). BiXGBoost: A scalable, flexible boosting‐based method for reconstructing gene regulatory networks. Bioinformatics, 35(11), 1893–1900. 30395189 10.1093/bioinformatics/bty908

[gepi22362-bib-0048] Zhou, W. , Fritsche, L. G. , Das, S. , Zhang, H. , Nielsen, J. B. , Holmen, O. L. , Chen, J. , Lin, M. , Elvestad, M. B. , Hveem, K. , Abecasis, G. R. , Kang, H. M. , & Willer, C. J. (2017). Improving power of association tests using multiple sets of imputed genotypes from distributed reference panels. Genetic Epidemiology, 41(8), 744–755. 28861891 10.1002/gepi.22067 PMC6324190

