# Supplementary material for: On the application, reporting, and sharing of in silico simulations for genetic studies
Source: Genet Epidemiol. 2020 Oct 16;45(2):131–41. doi: 10.1002/gepi.22362 (PMC7984380; doi:10.1002/gepi.22362)
Supplement: Supplementary file 1 — Supporting information. [file GEPI-45-131-s001.docx]

**Supplementary Table 1: List of Journals Surveyed**

| Journal | Volume/Issue | Ratio simulated | Percent simulated data |
| --- | --- | --- | --- |
| Genetic Epidemiology | 43/1 | 6/8 | 75% |
| Evolution | 63/12 | 9/20 | 45% |
| BMC Bioinformatics | 20/1 | 30/81 | 37% |
| Mol Biol Evol | 36/1 | 5/16 | 31% |
| Bioinformatics | 35/1 | 10/33 | 30% |
| Proc Biol Sci | 286/1894 | 7/27 | 26% |
| Nat Commun | 10/Jan | 25/100 | 25% |
| Genetics | 211/1 | 4/21 | 19% |
| Proc Natl Acad Sci U S A | 113/52 | 12/64 | 19% |
| Mol Ecol Resources | 19/1 | 4/22 | 18% |
| Nat Genet | 47/11 | 4/22 | 18% |
| Am J Hum Genet | 104/1 | 2/13 | 15% |
| Scientific Report | 8 | 50/350 | 14% |
| PLoS Genetics | 14/6 | 4/31 | 13% |
| Front Genet | 10 | 3/24 | 13% |
| PLoS One | 14/1 | 6/63 | 10% |
| BMC Genomics | 20/1 | 11/145 | 8% |

Journals that were selected for initial survey. We picked one issue from each journal, surveyed all articles in the issue, and determined the percent of articles that utilized genetic simulations.
